# Sequence characterization of the 5S ribosomal DNA and the internal transcribed spacer (ITS) region in four European *Donax* species (Bivalvia: Donacidae)

**DOI:** 10.1186/s12863-018-0684-x

**Published:** 2018-10-26

**Authors:** Jenyfer Fernández-Pérez, Ana Nantón, Josefina Méndez

**Affiliations:** Grupo Xenomar, Departamento de Bioloxía, Facultade de Ciencias and Centro de Investigaciones Científicas Avanzadas (CICA), Universidade da Coruña, Campus de A Zapateira, 15071 A Coruña, Spain

**Keywords:** *Donax*, Internal transcribed spacer, Ribosomal DNA, Wedge clams, 5S unit

## Abstract

**Background:**

The whole repeat unit of 5S rDNA and the internal transcribed spacer (ITS) of four European *Donax* species were analysed. After amplifying, cloning and sequencing several 5S and ITS units, their basic features and their variation were described. The phylogenetic usefulness of 5S and ITS sequences in the inference of evolutionary relationships among these wedge clams was also investigated.

**Results:**

The length of the 5S repeat presented little variation among species, except *D. trunculus* that differed from the rest of the *Donax* species in 170–210 bp. The deduced coding region covered 120 bp, and showed recognizable internal control regions (ICRs) involved in the transcription. The length of non-transcribed spacer region (NTS) ranged from 157 bp to 165 bp in *Donax trunculus* and from 335 bp to 367 bp in the other three species. The conservation degree of transcriptional regulatory regions was analysed revealing a conserved TATA-like box in the upstream region. Regarding ITS sequences, the four *Donax* species showed slight size differences among clones due to the variation occurring in the ITS1 and ITS2, except *Donax variegatus* did not display size differences in the ITS2. The total length of the ITS sequence ranged between 814 and 1014 bp. Resulting phylogenetic trees display that the two ribosomal DNA regions provide well-resolved phylogenies where the four European *Donax* species form a single clade receiving high support in nodes. The topology obtained with 5S sequences was in agreement with *Donax* evolutionary relationships inferred from several sequences of different nature in previous studies.

**Conclusions:**

This is not only a basic research work, where new data and new knowledge is provided about *Donax* species, but also have allowed the authentication of these wedge clams and offers future applications to provide other genetic resources.

**Electronic supplementary material:**

The online version of this article (10.1186/s12863-018-0684-x) contains supplementary material, which is available to authorized users.

## Introduction

In higher eukaryotes, rDNA comprises two different multigene families [53], including the major 45S rDNA family encoding 18S, 5.8S, and 28S rRNA, and the minor 5S rDNA family encoding 5S rRNA, each composed of hundreds to thousands of copies, organized in tandem repeats, and consisting of coding regions and transcribed and non-transcribed spacers. The different evolutionary rates among different regions, the secondary structure of these genes and their organization in tandem repeats, make rDNA attractive candidate for species identification, population characterization, phylogenetic studies and evolutionary relationships and genomic structure [39, 47, 77].

The 5S rDNA consists of a highly conserved 120 bp coding sequence (5S rRNA gene) clustered in long direct tandem arrays and separated by variable non-transcribed flanking DNA sequences know as non-transcribed spacers or NTSs. Both together, the coding sequence and the NTS, form a repeat unit that can be found in hundreds to thousands of copies tandemly repeated in the genome. Even though the 5S rRNA gene is highly conserved, even among unrelated species, the NTS are variable both in length and sequence. These discrepancies have been used as molecular phylogenetic and species-specific markers in several bivalve mollusc species [24, 26, 55, 72], so that the 5S rDNA is a good candidate to identify molecular markers suitable for distinguish related species.

By the same token, the internal transcribed spacer (ITS) region of rDNA consists of one coding region (5.8S rRNA gene) and two non-coding regions (ITS1 and ITS2) located in the rDNA between 18S and 5.8S rRNA genes and between 5.8S and 28S rRNA genes, respectively. Due to ITS sequences show more variability than their flanking coding region [38], they have been also frequently used to infer phylogenetic relationships among bivalve species [6, 45, 100] and to differentiate related bivalve species [39, 54, 86]. For instance, ITS1 has been a widely chosen marker for assessing variation within species due to its high level of divergence, while ITS2 region has been proposed as an effective barcode similar to the cytochrome c oxidase subunit I (COI) for identifying species that are difficult to distinguish morphologically and allowing identify closely related species within different families and genera [105].

To date, numerous reports on the characterization of the 5S rDNA and the ITS region in several molluscan species, including bivalves, gastropods, and cephalopods have been published [44, 56, 101]. But bivalve molluscs stand out for being one of the most extensively studied group of organisms regarding 5S rDNA and ITS region, showing high levels of gene organization as well as a vast diversity of gene arrangements. Molecular organization of ITS region and 5S rDNA has been studied in cockles [26–28, 43], mussels [44], oysters [9, 10], scallops [41, 42, 45, 46, 55], razor clams [25, 99, 100] and Veneroida clams [3], but it have never been studied in the wedge clams of the genus *Donax*.

Four *Donax* species, *Donax semistriatus*, *Donax trunculus*, *Donax variegatus* and *Donax vittatus*, are common along the European littoral and live sympatrically in some areas [1, 20, 21, 31, 84]. These marine bivalves play an important socioeconomic role in some European coastal regions. For instance, the wedge clam *D. trunculus* is an exploited and economically important traditional seafood in several European countries, including France [95], Italy [106], Portugal [4], Spain [61] and Turkey [69], which could emerge if managed properly on the local scale. However, natural beds of this species in Galicia (north – west coast of Spain) have been intensively exploited, and they have suffered a severe decrease. In fact, the amount harvested of this wedge clam has declined within the last 16 years from ~ 17 t (2001) to 171.10 kg (2017) (Consellería do Mar, Xunta de Galicia) and at present, only the fishermen’s association of Arousa (108.05 kg) and Cedeira (365.39 kg) commercialise this bivalve mollusc (data from [7]). On a much larger scale, only in the Iberian Peninsula, the recorded captures has suffered a sharp decline, with a maximum production of 1042 t in 2005, but reaching only 195 t in 2016 (FAO-FIGIS, 2018). In point of fact, currently some *D. trunculus* localities seem to be at high long-term risk of extinction [57]. Furthermore, *D. trunculus* may account for most of the recorded catches of FAO in these countries for genus *Donax*. Nevertheless, FAO statistics [18] do not distinguish between species. Therefore, it is likely to find in the fish market other *Donax* species with lower economic value being sold as *D. trunculus*. However, despite the economic importance that genus *Donax* has for the European seafood sector and of being an overexploited species, until a few years ago basic genetic studies in this organism were neglected. To date, with respect to the *Donax* genus, the complete female mitochondrial genomes have been sequenced and characterized [20] and three methods for the molecular identification of European *Donax* species have been developed [22, 65, 72]. Concerning *D. vittatus*, a work that deals with the description and study of the karyotype of *D. vittatus* and compares it with the karyotype of *D. trunculus* has recently been published [30] and its genetic diversity and population structure have been evaluated with mitochondrial and nuclear markers [21]. Regarding *D. trunculus*, most of the works have focused on studying the karyotype of the species [8, 34, 58], as well as on the analysis of mobile elements and satellite DNA [74, 75, 78–80, 87]. In addition, Theologidis et al. [96] have studied the mode of inheritance of mitochondrial DNA. Recently, microsatellite markers have also been developed [64, 82] and population genetic analyses based on this type of molecular markers [57, 63, 82] and mitochondrial markers [23] have been carried out.

In this work, the 5S rDNA and the ITS region of four wedge clams, *D. semistriatus*, *D. trunculus*, *D. variegatus* and *D. vittatus*, of the bivalve family Donacidae present in Europe were analysed. The aim of this analysis was to amplify, clone, and sequence the 5S rDNA and ITS repeats units to i) provide their basic characteristics, ii) assess their variability, iii) estimate their divergence and iv) report their utility in evolutionary relationships.

## Material and methods

### Sampling and DNA extraction

Twelve *Donax trunculus* specimens were collected from natural beds in Vilarrube (northwestern Spain) while twelve *D. semistriatus*, eight *D. variegatus* and twenty *D. vittatus* samples came from Portuguese coast (Table 1). Field work was conducted in accordance with local legislation and with regulations and guidelines established by the University of A Coruña. No endangered or protected species were involved. Specimens were taxonomically identified using a species-specific PCR-RFLP analysis of COI capable discriminating among the four *Donax* species [65]. Total genomic DNA was extracted from ethanol-preserved foot using a Chelex-100 (Sigma-Aldrich, USA) protocol based on Walsh et al. [102].

**Table 1:** Sampling details of species and GB Accession numbers

Species	Locality	Country	Coordinates		GB Accession numbers	
			Latitude	Longitude	5S rDNA	ITS
*D. semistriatus*	Monte Gordo	Portugal	37.167	−7.503	MG041608 - MG041634	MG041692 - MG041713
*D. trunculus*	Vilarrube	Spain	43.644	−8.077	MG041635 - MG041654	MG041714 - MG041736
*D. variegatus*	Monte Gordo	Portugal	37.100	−7.633	MG041655 - MG041676	MG041737 - MG041749
*D. vittatus*	Mira-Vagueira	Portugal	40.614	−8.769	MG041677 - MG041691	MG041750 - MG041761

### PCR amplification, cloning and sequencing

For 5S rDNA, amplification reactions were carried out using a set of primers designed by Fernández-Tajes and Méndez [24] annealing to the coding region in opposite orientations. They were carried out in 25 μl containing 150 ng of genomic DNA, 0.6 μM of each primer, 0.25 μM of each dNTP, 2 mM of MgCl_2_, 0.6 U of *Taq* polymerase (Roche Applied Science) and the buffer recommended by the polymerase suppliers. Cycling conditions were 2 min denaturing at 95 °C; (30 s at 95 °C, 30 s at 55 °C, and 1 min at 72 °C) × 35; and a final extension step at 72 °C for 5 min.

For ITS1 and ITS2, PCR reactions were performed with a pair of primers that anneal at the 3’end of the 18S ribosomal gene and the 5’end of the 28S ribosomal gene [37] (ITSF: 5′-GTTTCCGTAGGTGAACCTG-3′ and ITSR: 5´-CTCGTCTGATCTGAGGTCG-3′). They were performed in 25 μl containing 100 ng of genomic DNA, 0.25 μM of each dNTP, 1.5 mM of MgCl_2_, 1 μM of each primer, 0.625 U of *Taq* polymerase (Roche Applied Science) and the buffer recommended by the polymerase suppliers. Cycling conditions were 3 min denaturing at 94 °C; (20 s at 94 °C, 20 s at 55 °C, and 45 s at 72 °C) × 30; and a final extension step at 72 °C for 5 min. PCR products were migrated on a 2.0% agarose gel electrophoresis. Gels were stained by immersion in 0.5 μg/ml ethidium bromide solution for 30 min, visualized and recorded on a transilluminator Gel Doc XR Systems (Bio-Rad, Barcelona, Spain).

PCR products were migrated on 2% agarose gel electrophoresis. Gels were stained by immersion in 0.5 μg/ml ethidium bromide solution for 30 min, visualized and recorded on a transilluminator Gel Doc XR Systems (Bio-Rad, Barcelona, Spain).

For three or four individuals of each species, the product obtained was ligated into the T&A™ cloning vector and transformed into *Escherichia coli* ECOS™ JM109 strain competent cells using T&A™ Cloning Vector Kit (Yeastern Biotech Co., Ltd). Recombinant colonies were screened by PCR amplifying with M13 forward and reverse primers to assess the size of the insert. PCR reaction mixture contained 5 μl of recombinant cells, 1x PCR buffer, 1.5 mM MgCl_2_, 0.2 μM of each dNTP, 0.6 μM of each primer, and 0.3 U of *Taq* polymerase (Roche Applied Science) in a final volume of 12.5 μl. The thermal cycle profile consisted of an initial denaturation of 10 min at 94 °C, 30 cycles of 1 min at 94 °C, 1 min at 55 °C and 1 min at 72 °C; and a final extension of 10 min at 72 °C. Several recombinant colonies (3–10 per individual) were selected at random and grown in LB medium and, in order to purify the plasmids, a QIAprep Spin Miniprep Kit (QIAGEN) was used. Plasmids were sequenced using M13 primers (forward and reverse) on an ABI PRISM 3120xl (Applied Biosystems, Foster City, CA, USA) at the Molecular Biology Unit of the University of A Coruña (Spain). The corresponding nucleotide sequences have been deposited in the GenBank database under accession numbers MG041608 – MG041761 (Table 1).

### Sequence analysis

The identity of sequences obtained was corroborated using BLASTn searches of the NCBI database (https://blast.ncbi.nlm.nih.gov/Blast.cgi). Sequence data were aligned via MAFFT [50] using the L-INS-i algorithm (recommended for < 200 sequences with one conserved domain and long gaps) and manually checked using the BioEdit v.7.2.5 sequence editor [35]. The number of variable sites, nucleotide diversity and sequence divergence were estimated using DnaSP v5.10.01 [52]. Differences between sequence pairs and *Donax* consensus sequences were calculated using Geneious Pro v.4.8.5 [15]. For the phylogenetic analyses, sequence data were aligned in MAFFT [50] using the L-INS-i algorithm. 5S and ITS alignments, consisting of 652 and 1110 pb and including 27 sequences from *D. semistiatus*, 20 from *D. trunculus*, 21 from *D. variegatus*, 15 from *D. vittatus*, and 22 sequences from *D. semistiatus*, 23 from *D. trunculus*, 13 from *D. variegatus*, 12 from *D. vittatus*, respectively; and *Cerastoderma edule* (GB accession numbers: AJ132199.1 for 5S and AM229683.1 for ITS) and *Cerastoderma glaucum* (GB accession numbers: AJ842010.1 for 5S and AM229691.1 for ITS) like outgroups were then analysed using Bayesian Inference (BI). Akaike Information Criterion was selected using jModelTest v.2.1.8 [12] for each gene partition, including codon positions of coding genes. The optimal chosen method for 5S rDNA and ITS, were HKY + I + G and GTR + G, respectively. BI analyses were run using MrBayes v.3.2.6 [83]. Two independent Markov chain Monte Carlo (MCMC) runs, each comprising four linked chains (one cold and three heated; as default settings), were performed for 5,000,000 generations, sampling every 1000 generations to allow adequate time for convergence. The convergence of the two runs was assessed by stopping the analysis when the average standard deviation was below 0.01 (stoprule = yes and stopval = 0.01 in the mcmc command). 1,311,000 and 3189,000generations were enough to reach adequate average standard deviation (< 0.01) in 5S rDNA and ITS, respectively. The first 25% trees were discarded as burn-in.

## Results

### 5S rDNA

The 5S rDNA repeat unit was PCR amplified in at least 20 individuals of each wedge clam species, except for *D. semistriatus* and *D. variegatus* of which the number of available individuals was low (12 and 8, respectively). The length of the 5S units was about 275–300 bp for *D. trunculus*, about 450 bp for *D. variegatus* and around 500 bp for fragments obtained from the other two *Donax* species with minimal variation (1–12 bp) among clones (Table 2). Taking as reference the 5S rRNA sequences available in other bivalve species [9, 17, 25, 26, 44, 55], the coding region was assigned to 120 bp in the four cases and the non-transcribed spacer (NTS) region to the remaining sequence (Table 2). BLASTn analysis corroborated the identity of the limited region and indicated that no other coding sequence was included in the 5S rDNA repeat unit. The GC content of the repeat units ranged from 38.2 to 43.3% among the wedge clams (Table 2), with higher values in the coding region (53.4–55%) than in the spacer region (33.1–39.1%).

**Table 2:** Size (bp) and mean value of the GC content (%) of the 5S rDNA repeat unit

Species	No. of clones	Repeat unit		Coding region		NTS	
		Length	GC	Length	GC	Length	GC
*D. semistriatus*	27	475–487	40.5	120	54.6	355–367	35.9
*D. trunculus*	20	277–285	41.5	120	54.3	157–165	32.3
*D. variegatus*	22	455–456	43.3	120	55.0	335–336	39.1
*D. vittatus*	15	480–481	38.2	120	53.4	360–361	33.1

The alignment of all the 5S rDNA wedge clam sequences consisted of 568 pb and showed 130 variable sites of which 108 were parsimony informative, and 342 indels (due mainly to the fact that the sequence of *D. trunculus* is smaller than for the rest of the species). Almost entirely the variation was located in the spacer region; the sequence corresponding to gene showed 38 variable sites (Additional file 1). Intraindividual variation was minimal within control region and moderate within the spacer region in *D. trunculus* and *D. variegatus.* But in *D. semistriatus* and *D. vittatus* the sequences displayed considerable variation within the coding and spacer regions. For each species, in *D. semistriatus* the alignment of 27 clones showed 139 variable sites (17 nucleotide substitutions located in the coding region and 122 in the NTS) and 29 indels located in the NTS. The percentage of differences in pairwise comparisons ranged from 5.2 to 7.5% in intraindividual comparisons and from 6.3 to 7.3% in interindividual comparisons. Global nucleotide diversity was 0.05744 (0.01360 in 5S and 0.07342 in NTS). In *D. trunculus* the alignment of 20 clones showed 25 variable sites (6 nucleotide substitutions located in the coding region and 19 in the NTS; and 11 indels located in the NTS). The percentage of differences in pairwise comparisons ranged from 2.0 to 2.8% in intraindividual comparisons and from 2.5 to 3.3% in interindividual comparisons. Global nucleotide diversity was 0.02176 (0.01948 in 5S and 0.02359 in NTS). In the case of *D. variegatus* the alignment of 22 clones presented 13 variable sites (4 nucleotide substitutions located in the coding region and 9 in the NTS) and an indel located in the NTS. Only four clones (*Dvar*1/5, *Dvar*1/8, *Dvar*2/3 and *Dvar*4/3) displayed a nucleotide substitution in the coding region. The percentage of differences in pairwise comparisons ranged from 0.1 to 0.4% in intraindividual comparisons and from 0.2 to 0.6% in interindividual comparisons. Overall nucleotide diversity was 0.00393 (0.00305 in 5S and 0.00425 in NTS). In *D. vittatus* the alignment of 15 clones displayed 69 variable sites (15 nucleotide substitutions located in the coding region and 54 in the NTS) and an indel located in the NTS. The percentage of differences in pairwise comparisons ranged from 0.0 to 2.4% in intraindividual comparisons and from 3.2 to 5.3% in interindividual comparisons. Overall nucleotide diversity was 0.05607 (0.04828 in 5S and 0.05881 in NTS). The values of nucleotide divergence (*D*_*xy*_) and the net number of nucleotide substitutions between groups (*D*_*a*_) with Jukes and Cantor [48] method are shown in Table 3. The values obtained between species are similar, with the highest values being found between *D. variegatus* and *D. vittatus*, and lower between *D. semistriatus* and *D. vittatus*. These results are in agreement with the 5S phylogenetic tree (see below).

**Table 3:** *D*_*a*_ (above diagonal) and *D*_*xy*_ values (below diagonal) and their standard deviation in the four *Donax* species analysed. The values of the diagonal correspond to the values of nucleotide diversity (π) of the 5S rDNA repeat unit

	*D. semistriatus*	*D. trunculus*	*D. variegatus*	*D. vittatus*
*D. semistriatus*	0.05744	0.31435 ± 0.02371	0.53230 ± 0.05356	0.13307 ± 0.01849
*D. trunculus*	0.35038 ± 0.02368	0.02176	0.28505 ± 0.04513	0.36153 ± 0.04897
*D. variegatus*	0.56132 ± 0.05355	0.29773 ± 0.04512	0.00393	0.65667 ± 0.09996
*D. vittatus*	0.18950 ± 0.01841	0.39845 ± 0.04895	0.68411 ± 0.09994	0.05607

*D*_*xy*_ denotes the average number of substitutions per site between species and *D*_*a*_ the number of net substitutions between species [67]

**Fig. 1:**
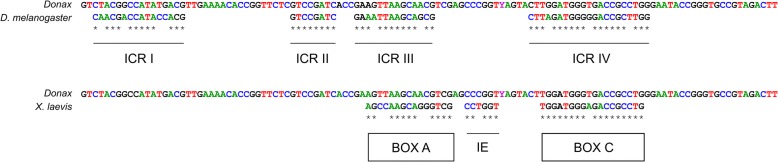
Graphical representation of the control elements involved in the transcription of 5S rDNA. The top sequences represent a schematic comparison of the ICRs between *Donax* consensus sequence and *D. melanogaster.* The bottom sequences represent a schematic comparison of the sequence elements (box A, intermediate element (IE), and box C) between *Donax* consensus sequence and *X. laevis.* The asterisks indicate similarities respect to consensus sequences described for *D. melanosgaster* and *X. laevis*

In the four *Donax* species, the internal control regions (ICRs) described in other organisms were identified. A graphical representation of the 5S internal promoters and their consensus sequences is shown in Fig. 1. The stretches from 3 to 18, from 37 to 44, from 48 to 61, and from 78 to 98 in the alignment displayed high homology with their orthologues ICR I, II, III, and IV of *Drosophila melanogaster* [88] (12/16, 8/8, 12/14, and 18/21 matches, respectively) (see Fig. 1), and the stretches from 50 to 64, from 67 to 72, and from 80 to 97 were also similar to box A, intermediate element, and box C of *Xenopus laevis* somatic 5S RNA gene [76] (11/15, 5/6, and 17/18 matches, respectively) (see Fig. 1). Moreover, the NTS region of *Donax* species contain TATA-like motif recognized at around − 28 nucleotides, other potential transcription control sequences that may be involved in 5S transcription initiation, such as in the silkworm *Bombyx mori* [62], in *Neurospora crassa* [98] and *D. melanogaster* [88]. The NTS sequences of *D. semistriatus* and *D. vittatus* retained the complete block TATATA at the 3’end; but not the other species, *D. trunculus* and *D. variegatus*, because one insertion T(G)ATATA and a point mutation (TATTTA) occurred within, respectively. Finally, a T-rich stretch was located a few residues downstream of the coding region in the four *Donax* species, and it is believed that could be related to transcriptional processes, specifically in transcription termination [2, 32, 40].

### ITS1 and ITS2

The ITS region was PCR amplified in at least 20 individuals of *D. trunculus* and *D. vittatus*, and 12 individuals of *D. semistriatus* and 8 individuals of *D. variegatus*, yielded a single product about 1000 bp for *D. vittatus*, about 800 bp for *D. trunculus* and about 900 bp for the rest of species. Table 4 shows the size and GC of the ITS region delimited according to the BLASTn analysis result. The four *Donax* species showed slight size differences among clones due to the variation occurring in the ITS1 and ITS2, except *D. variegatus* did not display size differences in the ITS2. The total length of the ITS region was 814–1014 bp with 58–62.4% GC content; ITS1 was 400–542 bp and 59.7–62.6% GC; the 5.8S rRNA gene was 157 bp and 57.3% GC in all clones; and ITS2 was 254–316 bp and 55.7–64.8% GC. The GC content was similar in the four species, with the highest values in the ITS2 followed ITS1 and 5.8S gene, except for *D. trunculus* that displayed higher content in ITS1 than in ITS2 (Table 4).

**Table 4:** Size (bp) and mean value of the GC content (%) of the ITS region

Species	No. of clones	ITS1		5.8 gene		ITS2		ITS	
		Length	GC	Length	GC	Length	GC	Length	GC
*D. semistriatus*	22	452–457	62.6	157	57.3	283–287	64.0	892–900	62.1
*D. trunculus*	23	400–405	59.7	157	57.3	254–269	55.7	814–828	58.0
*D. variegatus*	13	452–453	62.6	157	57.3	283	64.8	892–893	62.4
*D. vittatus*	12	534–542	61.0	157	57.3	307–316	61.1	998–1014	60.5

The alignment of the different *Donax* ITS sequences consisted of 1048 pb and showed 258 variable sites of which 248 were parsimony informative sites, and 279 indels (see in Additional file 2). The largest differences were found in ITS1 (162 variable sites), followed by ITS2 (90 variable sites) and 5.8 gene (6 variable sites). For each species, in *D. semistriatus* the alignment of 22 clones showed 33 variable sites (20 nucleotide substitutions located in the ITS1, one in the 5.8 gene and 12 in the ITS2) and 6 and 4 indels located in the ITS1 and ITS2, respectively. The percentage of differences in pairwise comparisons ranged from 0.2 to 2.5% in intraindividual comparisons and from 1.7 to 3.7% in interindividual comparisons. Overall nucleotide diversity was 0.01415 (0.01827 in ITS1, 0.00323 in ITS2 and 0.00058 in 5.8S). In *D. trunculus* the alignment of 23 clones presented 53 variable sites (21 nucleotide substitutions located in the ITS1, 2 in the 5.8 gene and 30 in the ITS2) and 7 and 17 indels located in the ITS1 and ITS2, respectively. The percentage of differences in pairwise comparisons ranged from 0.0 to 1.3% in intraindividual comparisons and from 0.03 to 2.1% in interindividual comparisons. Global nucleotide diversity was 0.01923 (0.01229 in ITS1, 0.04105 in ITS2 and 0.00314 in 5.8S). In *D. variegatus* the alignment of 13 clones displayed 28 variable sites (22 nucleotide substitutions located in the ITS1, and 6 in the ITS2) and 4 indels located the ITS1. The percentage of differences in pairwise comparisons ranged from 0.00 to 0.01% in intraindividual comparisons and from 0.04 to 1.80% in interindividual comparisons. Overall nucleotide diversity was 0.01225 (0.01998 in ITS1, 0.00703 in ITS2 and 0.00000 in 5.8S). In *D. vittatus* the alignment of 12 clones showed 59 variable sites (17 nucleotide substitutions located in the ITS1, one in the 5.8 gene and 41 in the ITS2) and 11 and 38 indels located the ITS1 and ITS2, respectively. The percentage of differences in pairwise comparisons ranged from 0.2 to 2.20% in intraindividual comparisons and from 2.7 to 3.6% in interindividual comparisons. Global nucleotide diversity was 0.02166 (0.01272 in ITS1, 0.05163 in ITS2 and 0.00107 in 5.8S). In addition, the alignment of the four species revealed four stretches of 12, 23, 13, and 59 nucleotides in ITS1 (alignment positions 241–252, 316–338, 387–399 and 499–557, respectively) and four stretches of 17, 33, 22 and 17 nucleotides in ITS2 (alignment positions 715–731, 754–786, 790–811 and 862–878, respectively), all being highly conserved among *Donax* species (percentages of similarity higher than 96.7%). Sequence similarity of ITS2 (76.4%) was higher than that of ITS1 (70.6%) across species. The values of nucleotide divergence (*D*_*xy*_) and the net number of nucleotide substitutions between groups (*D*_*a*_) with Jukes and Cantor [48] method are shown in Table 5. The highest values being found between *D. trunculus* and *D. vittatus*, and lower between *D. semistriatus* and *D. variegatus*. These results are in agreement with the phylogenetic tree derived from the ITS region (see below).

**Table 5:** *D*_*a*_ (above diagonal) and *D*_*xy*_ values (below diagonal) and their standard deviation in the four *Donax* species analysed. The values of the diagonal correspond to the values of nucleotide diversity (π) of the ITS region

	*D. semistriatus*	*D. trunculus*	*D. variegatus*	*D. vittatus*
*D. semistriatus*	0.01415	0.30096 ± 0.03807	0.00386 ± 0.00439	0.09768 ± 0.01797
*D. trunculus*	0.31946 ± 0.03804	0.01923	0.30675 ± 0.04396	0.32262 ± 0.04058
*D. variegatus*	0.01805 ± 0.00459	0.32206 ± 0.04394	0.01225	0.09384 ± 0.02086
*D. vittatus*	0.11563 ± 0.01780	0.33954 ± 0.04054	0.11027 ± 0.02065	0.02166

*D*_*xy*_ denotes the average number of substitutions per site between species and *D*_*a*_ the number of net substitutions between species [67]

### Phylogenetic analyses

**Fig. 2:**
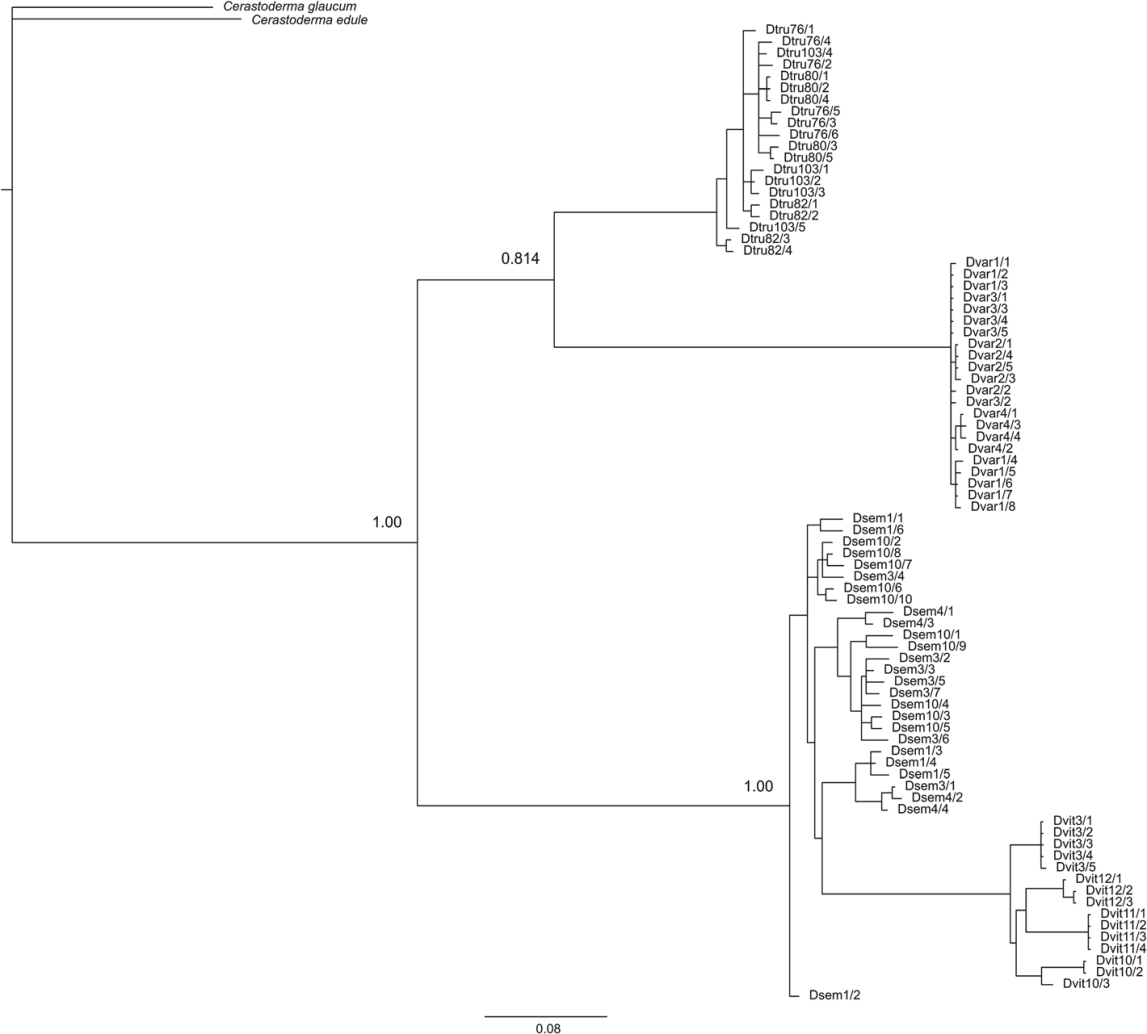
Bayesian phylogenetic tree inferred from 5S rDNA sequences of *Donax semistriatus*, *Donax trunculus*, *Donax variegatus* and *Donax vittatus*. The phylogenetic tree was rooted with *Cerastoderma edule* and *Cerastoderma glaucum* species. Numbers at the nodes correspond to Bayesian posterior probabilities

**Fig. 3:**
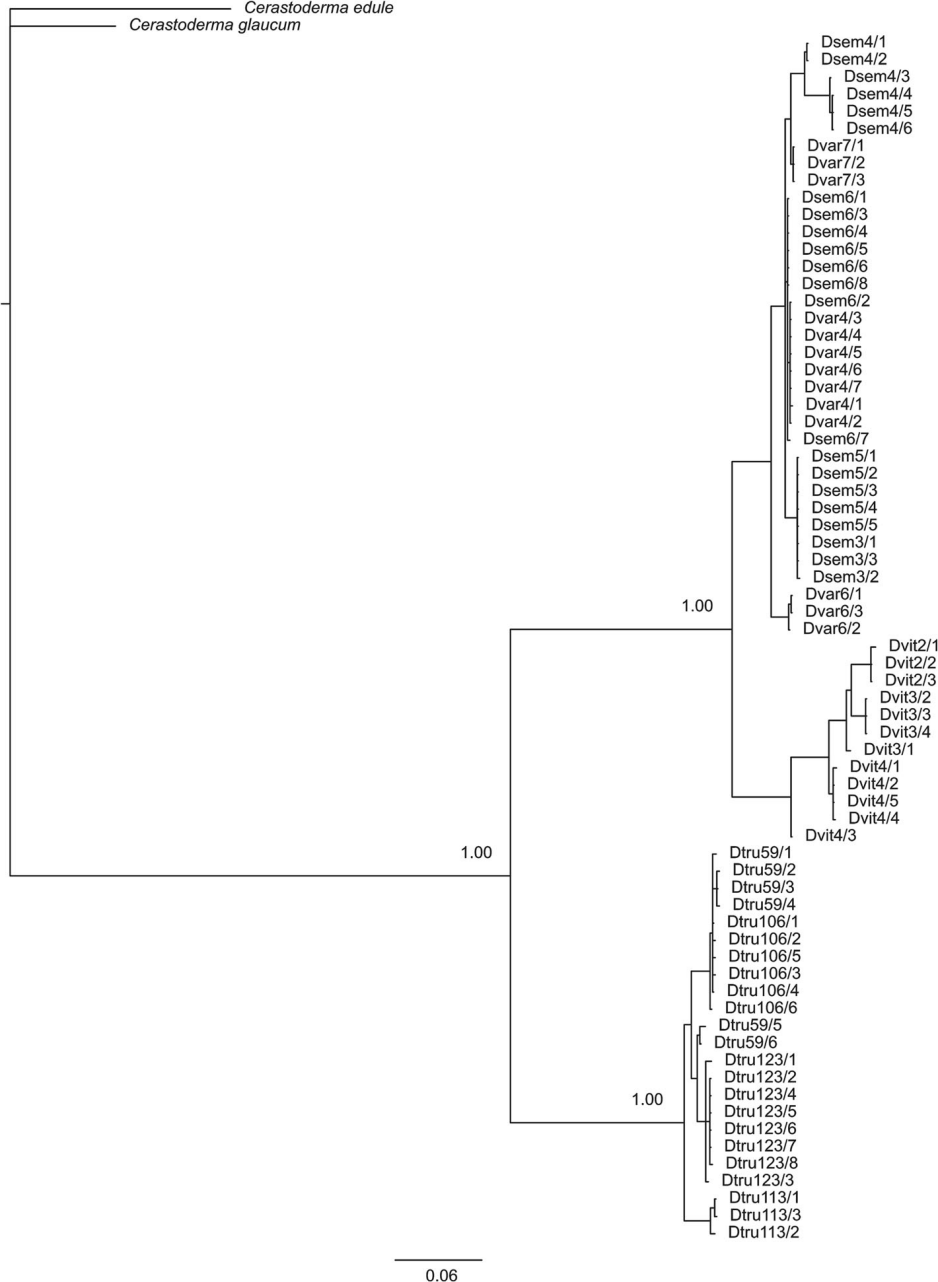
Bayesian phylogenetic tree inferred from ITS sequences of *Donax semistriatus*, *Donax trunculus*, *Donax variegatus* and *Donax vittatus*. The phylogenetic tree was rooted with *Cerastoderma edule* and *Cerastoderma glaucum* species. Numbers at the nodes correspond to Bayesian posterior probabilities

Regarding phylogenetic analyses, the results showed well-resolved phylogenies where the four *Donax* species form a single clade and received high Bayesian support values in nodes (Figs. 2 and 3). However, 5S and ITS tree topologies were not congruent. On the one hand, the BI tree inferred from 5S rDNA sequences of the four *Donax* species (Fig. 2) shows two groups supported by high posterior probabilities, where *D. trunculus* + *D. variegatus* is the sister clade of *D. semistriatus* + *D. vittatus*. These results agree with the values of *D*_*xy*_ and *D*_*a*_ showed in Table 3, where the highest values being found between *D. variegatus* and *D. vittatus*, and lower between *D. semistriatus* and *D. vittatus.* On the other hand, the BI tree from ITS sequences (Fig. 3) consisted of two well-supported (with 1.00 posterior probability as branch support) sister clades: one comprising solely *D. trunculus* sequences, and the other including the remaining *Donax* ones, where *D. semistriatus* and *D. variegatus* appear in the same branch. These results are also in accordance with the values of *D*_*xy*_ and *D*_*a*_ showed in Table 5, where the highest values being found between *D. trunculus* and *D. vittatus*, and lower between *D. semistriatus* and *D. variegatus*.

## Discussion

This work provides the nucleotide sequences of the 5S rDNA and the ITS region of four European *Donax* species, describes and characterizes for the first time in this group of organisms the general characteristics of these sequences and analyses their variation.

Regarding the 5S rDNA of the four wedge clams studied show, at least in part, the conventional tandem arrangement, as deduced from successful amplification using contiguous primers with opposite orientation. Moreover, the BLASTn analysis showed that the 5S rRNA genes are separated from one another by a NTS region; the coding region was assigned to 120 bp in the four species and the non-coding spacer to the remaining sequence. The length of the characterized repeat units presented little variation among species (5–32 bp), except *D. trunculus* with a repeat unit of 277–285 bp and differing from the rest of the *Donax* species in 170–210 bp. Compared to other bivalve species, the *Donax* 5S rDNA units are among the shortest with the scallops *Aequipecten opercularis* (433–465 bp), *Mimachlamys varia* (453–455 bp), *Hinnites distortus* (451 bp) and *Pecten maximus* (463 bp) [42, 55] and the razor clams *Ensis arcuatus* (420 bp), *Ensis siliqua* (422 bp), *Ensis directus* (443 bp) and *Ensis macha* (434 bp) [24]; although the cockles *C. edule* and *C. glaucum* have a repeat unit of 544–546 and 539–568 bp [26, 43], respectively; the oysters about 1100 bp in *Crassostrea* and 2000 bp in *Ostrea* [9, 10]; and the mussels *Mytilus edulis*, *Mytilus galloprovincialis* and *Mytilus trossulus* have three types,~260 bp (α band), ~770 bp (β band) and ~1000 bp (γ band) in length, the mussel *M. californianus* has other three types ~240 bp (small-β band), ~730 bp (β band) and ~980 bp (γ band), and the mussel *M. coruscus* has a repeat unit of ~300 bp [27]. These differences in size are due to the NTS and have been very useful to differentiate among wedge clams and from other bivalves when morphological criteria are difficult, for instance processed samples, samples without shell or during the larval stage [22, 72]. Despite the fact that *Donax* species display length differences in the spacer region and a high sequence divergence deduced from the difficulty in obtaining unambiguous sequence alignments, all four species studied show similar GC content (38.20–43.35%), with higher values in the gene (53.40–55.00%) than in the NTS (33.10–39.10%). This difference between coding and spacer region not only occurs in several bivalve species previously studied, such as cockles, mussels and scallops [26, 27, 44, 55], but also in species of crustaceans [70] and fish [85, 90], which have AT rich spacers (73% and > 57%, respectively). On the contrary, the mammalian 5S NTS has been shown to be GC rich (> 60%) [91, 92], and the spacer of the oocyte-type 5S rDNA of *Xenopus* is AT rich but that of the somatic type is GC rich [73].

The four *Donax* 5S rDNA units consisted of a coding region linked to a spacer without any other coding sequence associated. This result agree with that observed in cockles [26] and scallops [55], but contrasts with alternative arrangements that have been described, such as the linkage between 5S rDNA and small nuclear RNA (snRNA) in the crustacean *Asellus aquaticus* [71], the oysters *Crassostrea gigas* and *Crassostrea angulata* [9] and the sole *Solea senegalensis* [56], or the linkage of 5S gene with histone genes in the mussel *M. galloprovincialis* [16]. Although the use of other PCR amplification conditions or the analysis of a genomic library could reveal 5S rDNA units linked to other multigene families, it is not surprising that the *Donax* 5S rDNA arrangement differ from other bivalve or species, as the 5S rRNA gene linkages seem to be repeatedly established and lost during the evolution of eukaryotic genomes [14].

The ICRs involved in the transcription of 5S rDNA and the sequence elements box A, IE, and box C were identified in the four *Donax* species (Fig. 1). Furthermore, all 5S rDNA sequences showed TATA-like motifs upstream of the coding region and they were very similar to that reported in *B. mori* [62], in *N. crassa* [98], *D. melanogaster* [88] and several fish species [59]. Although functional assays are necessary to know the role of these TATA-like motifs, according to the position, they are good candidates for the interaction with TFIIIB for being located near the gene [26]. Recently, Raha et al. [81] have proposed that this region could be involved in RNA pol III transcription together with RNA pol II-like transcriptional factors. Nevertheless, it was less conserved in *D. trunculus* and *D. variegatus* because one insertion (T(G)ATATA) and a point mutation (TATTTA) occurred within, respectively, as it happens in cockle, razor clam and scallop species [101].Therefore, as previous authors indicate [101], this could imply that in these molluscan groups i) the 5S rDNA transcription could not precisely be regulated by RNA pol II–like transcriptional factors, ii) they could present lower transcriptional activities, or iii) they do not require the same level of sequence specificity. Moreover, all 5S rDNA sequences displayed a T-rich stretch potentially related to transcription termination [2, 32, 40].

Regarding the ITS region, the lengths determined for both ITS1 (400–542 bp) and ITS2 (254–316 bp) in the four *Donax* species are in line with those of other bivalve species. Average ITS1 length was 461.9 bp, and GC content was 61.5%, values very similar to those obtained by Chow et al. [5], who studied the ITS1 of several marine animals and reported wide data regarding length and GC content for marine mollusc species (in Mollusca average ITS length was 492.5 bp, and GC content was 55.9%). The ITS1 and ITS2 lengths for the clams *Venerupis pullastra*, *Ruditapes decussatus* and *Ruditapes philippinarum* ranged between 600 and 715 and 316–396 pb, respectively [19]. The ITS1 and ITS2 of four scallops (*A. opercularis*, *M. varia*, *H. distortus*, and *P. maximus*) are 209–277 and 270–294 bp [45, 103], respectively; and their GC content was 43–49% and 44–49%. They ranged between 367 and 514 and 317–446 bp in the Unionoidea species *Unio pictorum*, *Unio tumidus*, *Unio crassus*, *Anodonta anatina*, *Anodonta cygnea*, *Pseudanodonta complanata*, and *Margaritifera margaritifera* [49]. In the Veneridae species *Meretrix meretrix*, *Cyclina sinensis*, *Mercenaria mercenaria*, *Protothaca jedoensis*, *Dosinia corrugata* and *R. philippinarum*, ITS1 and ITS2 length were 522–900 and 281–412 bp, respectively; and their GC content were 57.66–65.62% and 65.21–67.87% [3]. In the two cockles *C. edule* and *C. glaucum*, ITS1 and ITS2 length ranged between 226 and 251 and 305–325 bp, and their GC content was 52–62% and 61–63%, respectively [28]. Data on ITS1 and ITS2 length in the razor shell *E. directus* ranged between 484 and 510 and 295–299 bp, and their average GC content were 58.9% for ITS1 and 63% for ITS2 [100]. Thus, *Donax* species ITS length and GC content were similar to those found in other bivalve species. Just as in the clams *V. pullastra*, *R. decussatus* and *R. philippinarum* [19] and other Veneridae species [3], the ITS1 *Donax* spacer is longer than ITS2, while in other bivalves the two spacers differ by < 100 bp [28, 49, 51]. As bivalve data accumulate, it seems that there are few restrictions that affect the variation in spacer length, since any type of the following situations may occur: ITS1 and ITS2 of similar size, ITS1 longer than ITS2 and ITS2 longer than ITS1 [28]. ITS GC content in *Donax* species is similar to that in venerids, *E. directus* and *Cerastoderma* species, as would be expected considering that scallops are Pteriomorphia bivalves, and venerids, *Ensis* and *Cerastoderma* species are Heterodonta. The high GC content of the ITS1 and ITS2 contrasted with the very low GC content of the NTS. This could be due to the fact the NTS region is not transcribed or folded into a secondary structure, whereas both ITS1 and ITS2 are transcribed and have known secondary structures. Maybe the high GC content is related to secondary structure stability. The length showed here for the 5.8S gene (157 bp) was previously described for the ocean quahog *Arctica islandica* [11], the four scallops studied by Insua et al. [45], and the six Veneridae species studied by Cheng et al. [3]; although sizes of 158 bp [19], 156 bp [51], and 158–161 bp [28] were reported in some species, but all of them are in line with the average length of eukaryote 5.8S rRNA of about 160 bp deduced from direct sequencing [66]. As expected for a high conserved sequence, the GC content of the 5.8S gene did not show variation (57.3%) and the values correspond to those observed in bivalves [28] and other animals [68, 94, 104].

The four *Donax* species showed intraindividual variation mainly in the spacers, ITS1 being more variable than ITS2 in *D. semistriatus* and *D. variegatus*, as evidenced by the number of variable sites in the sequence alignments and the distance values in pairwise comparisons. This is in line with that observed in other organisms such as *Drosophila* [89], *Similium damnosum* [93], and *Cerastoderma* [28]. By the contrast, ITS2 being more variable than ITS1 in *D. trunculus* and *D. vittatus*, as in scallop species [45] and with similar values to that described in ITS2 in the pearl oysters *Pinctada martensi*, *Pinctada maxima*, *Pinctada margaritifera*, *Pinctada chemnitzi*, *Pinctada nigra*, and *Pteria penguin* [36]. Nevertheless, intraindividual variation of the ITS sequences for *D. variegatus* was minimal or almost nonexistent as in the *M. varia* and *P. maximus* scallop species [45]. Therefore, it seems that intradindividual variation in *D. variagatus* is more moderate than that showed in the other three *Donax* species and that described in some other animal species [29, 68, 93, 104]. Globally, both ITS1 and ITS2 show sequence variation among wedge clams, with sequence similarity of ITS2 higher than that of ITS1 across species. However, blocks highly conserved across the *Donax* species were distinguished both in ITS1 and ITS2, which may suggest that they play a role in rRNA processing.

Overall, 5S and ITS sequences show higher values of nucleotide diversity (*D. trunculus*: 0.019–0.022; *D. vittatus*: 0.022–0.056) than other nuclear (18S, 28S and H3) and mitochondrial (16S and Cytb) markers in the same *Donax* species (*D. trunculus*: ~0.005, [23]; *D. vittatus*: 0–0.007, [23]), possibly due to the high variability of NTS and ITS, even though 5S and 5.8S genes present high conservation degree through species [14, 38], but also they are of smaller length. These results are in line with other marine species such as *Hexaplex trunculus* [33] where 5S was more variable than mitochondrial sequences (12S, 16S and COI).

The phylogenetic analyses inferred from 5S rDNA sequences provides a similar tree to that based on the 13 protein-coding genes of mitochondrial genome of the same species [20], the phylogeny based on several mitochondrial (16S, COI and Cytb) and nuclear (18S, 28S and H3) genes [21], and the phylogenetic tree derived from the mitochondrial COI gene [30]. This is in accordance with other bivalve studies where phylogenies have been successfully reconstructed by using the 5S region (e.g. [55, 101]). On the other hand, ITS phylogeny displays a different topology, but in all cases *D. semistriatus* and *D. vittatus* species are grouped in the same clade when markers of different nature are used [20, 21, 30]. In a previous study carried out by Chow et al. [5] who studied the ITS1 in several marine animals was reported that ITS1 has a limited utility for phylogenetic analysis. Anyway, phylogenies based on larger genetic regions, for instance mitogenomes, should thus be preferred.

Due to the variation observed in the 5S rDNA and ITS region among *Donax* species, these sequences have allowed the identification of reliable molecular markers that have been used to differentiate these wedge clams [22]. In this way, it has possible to develop a proper tool, based on multiplex PCR, which could be easily implemented by the government or private entities to guarantee the correct identification and authentication of commercial seafood products avoiding the unintentional substitution of different wedge clam, or detecting and avoiding fraud, to ensure composition and safety of commercial marine products, to protect consumers’ rights and to achieve other quality objectives, such as a certificate of origin [22]. Furthermore, this technique could be useful for conservation of these marine resources and species differentiation to obtain seed with correct identity [22]. In fact, the use of the 5S rDNA and ITS have been reported to be useful for discrimination of several bivalve species with commercial value, such as clams [19, 39], cockles [26], mussels [13, 37, 86, 97], oysters [10], razor clams [24], scallops [54] and wedge clams [72]. Additionally, these sequences could be studied to provide other genetic resources allowing to undertake further molecular and cytogenetic studies of this important bivalve species. For instance, 5S and ITS sequences could be used as probes in fluorescent in situ hybridization (FISH) experiments to study the possibility of hybridization in four *Donax* species studied here due to the fact that these species can live on the same beds. These sequences have been studied in the oysters *Pinctada fucata* and *Pinctada maculata* [60] and in the clams *R. decussatus* and *R. philippinarum* [39] for this same purpose.

## Conclusions

This is not only a basic research work, where we describe and characterize, for the first time, the 5S rDNA and the ITS regions in four bivalve molluscs belonging to the genus *Donax*, but also new data and new knowledge is provided for the scientific community about *Donax* species. Moreover, sequences provided here have allow to develop a method for authentication of the four European *Donax* species, and they will allow to undertake further genetic studies.

## Additional files

Additional file 1: Alignment of the 5S rDNA sequences of the four European *Donax* species. (FAS 49 kb)

Additional file 2: Alignment of the ITS sequences of the four European *Donax* species. (FAS 74 kb)

## Abbreviations

COI: Cytochrome c oxidase subunit I
ICRs: Internal control regions
ITS: Internal transcribed spacer
NTS: Non-transcribed spacer
RFLPs: Restriction Fragment Length Polymorphisms
